# Organoid technology and applications in cancer research

**DOI:** 10.1186/s13045-018-0662-9

**Published:** 2018-09-15

**Authors:** Hanxiao Xu, Xiaodong Lyu, Ming Yi, Weiheng Zhao, Yongping Song, Kongming Wu

**Affiliations:** 1 Department of Oncology, Tongji Hospital of Tongji Medical College, Huazhong University of Science and Technology, 1095 Jiefang Avenue, Wuhan, 430030 Hubei China; 2 Central Laboratory, the Affiliated Cancer Hospital of Zhengzhou University, Henan Cancer Hospital, Zhengzhou, 450000 Henan China; 3 Department of Hematology, the Affiliated Cancer Hospital of Zhengzhou University, Henan Cancer Hospital, Zhengzhou, 450000 Henan China

**Keywords:** Organoid, Cancer, Drug development, Drug efficacy, Drug toxicity, Personalized medicine, Immunotherapy, Regeneration medicine

## Abstract

During the past decade, the three-dimensional organoid technology has sprung up and become more and more popular among researchers. Organoids are the miniatures of in vivo tissues and organs, and faithfully recapitulate the architectures and distinctive functions of a specific organ.

These amazing three-dimensional constructs represent a promising, near-physiological model for human cancers, and tremendously support diverse potential applications in cancer research. Up to now, highly efficient establishment of organoids can be achieved from both normal and malignant tissues of patients. Using this bioengineered platform, the links of infection-cancer progression and mutation-carcinogenesis are feasible to be modeled. Another potential application is that organoid technology facilitates drug testing and guides personalized therapy. Although organoids still fail to model immune system accurately, co-cultures of organoids and lymphocytes have been reported in several studies, bringing hope for further application of this technology in immunotherapy. In addition, the potential value in regeneration medicine might be another paramount branch of organoid technology, which might refine current transplantation therapy through the replacement of irreversibly progressively diseased organs with isogenic healthy organoids.

In conclusion, organoids represent an excellent preclinical model for human tumors, promoting the translation from basic cancer research to clinical practice. In this review, we outline organoid technology and summarize its applications in cancer research.

## Background

During the past decades, enormous efforts have been exerted to cancer research [1, 2] and substantial progresses have been achieved in diagnosis [3, 4] and treatment [5–12]. However, cancer still represents a major worldwide health concern and many obstacles remain to be solved for further improving life quality and prolonging survival of cancer patients. The development of effective treatment regimens is among the major hurdles. Due to poor recapitulation of human tumors by conventional cancer models, numerous drugs working in these cancer models are finally eliminated in clinical trials because of either ineffectiveness or unbearable side effects.

Traditional two-dimensional (2D) cell line cultures and patient-derived tumor xenografts (PDTXs) have long been employed as tumor models and have made tremendous contribution to cancer research. However, many drawbacks hamper these two models for clinical use. 2D cell line cultures show their inability in simulating some vital subjects, such as the immune system, microenvironment, stromal compartments, and organ-specific functions. Other limitations include the lack of genetic heterogeneity of original tumors after many passages for cancer cell lines [13] as well as experiencing mouse-specific tumor evolution [14] and being consuming in money, time, and resources for PDTXs [15].

**Fig. 1:**
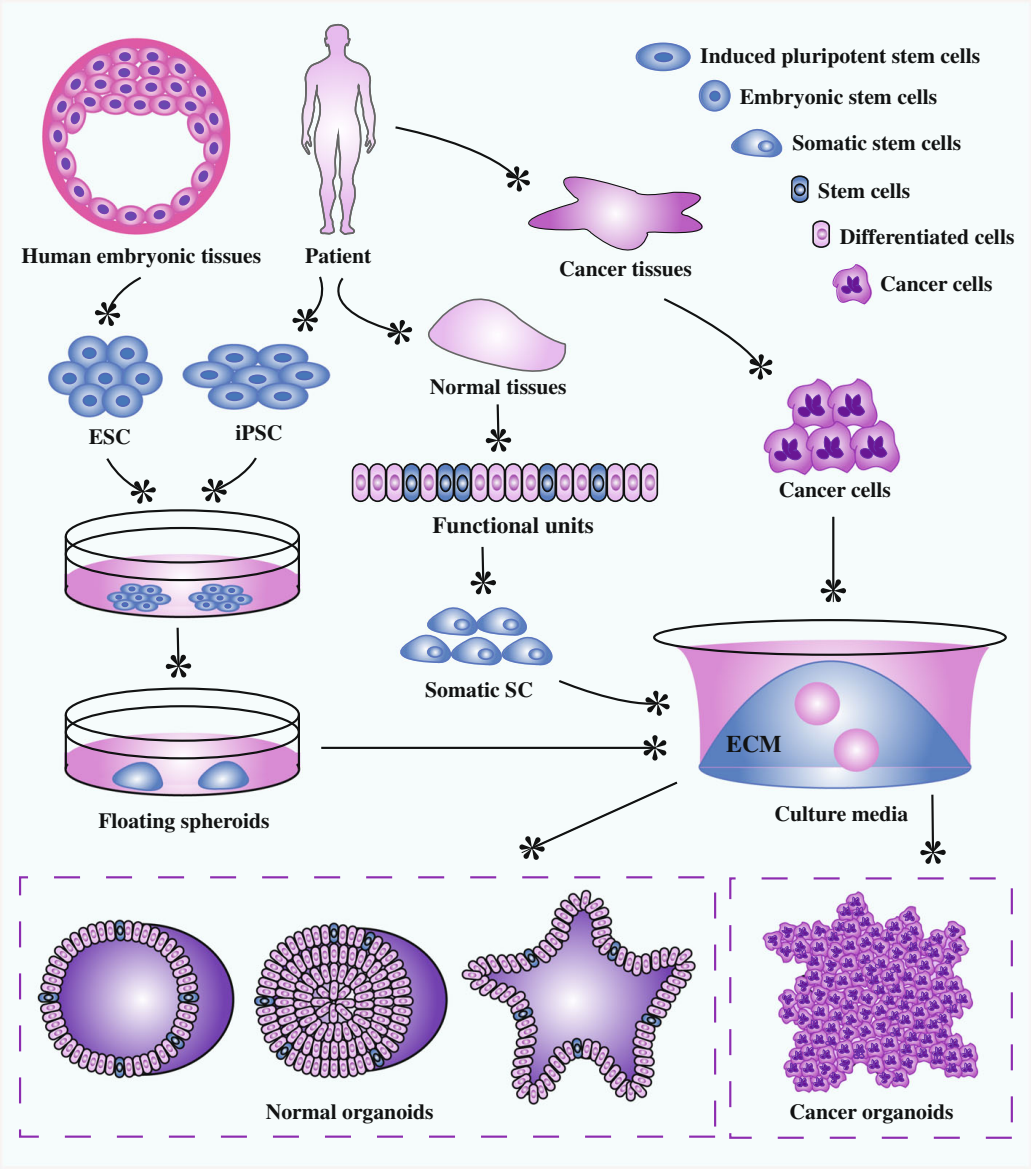
Organoid establishment from stem cells and cancer cells. Embryonic stem cells from human embryonic tissues and induced pluripotent stem cells from adult tissues firstly experience directed differentiation, generate floating spheroids, and subsequently are planted on extracellular matrix in specific culture medium to initiate organoid culture. Primary tissues from patients can be dissociated into functional units, which contain somatic stem cells. These somatic stem cells are enriched and cultured in three-dimensional medium to form organoids. Tumor cells isolated from cancer tissues can also form tumoroids in well-defined three-dimensional culture

Organoid technology springs up and becomes an independent research tool. Organoids are three-dimensional (3D) constructs and can be developed from embryonic stem cells (ESCs), induced pluripotent stem cells (iPSCs), somatic SCs, and cancer cells in specific 3D culture system (Fig. 1). Stem cells are a class of under-differentiated cells with self-renewing capacity and the potential to regenerate various tissues and organs. According to the developmental stage in which stem cells are located, they are divided into embryonic stem cells and adult stem cells. Embryonic stem cells are a type of cells isolated from early embryos with the ability of unlimited proliferation, self-renewal, and multi-directional differentiation. Progenitor cells belong to adult stem cells and are undifferentiated pluripotent or multipotent stem cells. Progenitor cells are present in various adult tissues of organisms and are responsible for the repair and regeneration process after tissue damage.

**Fig. 2:**
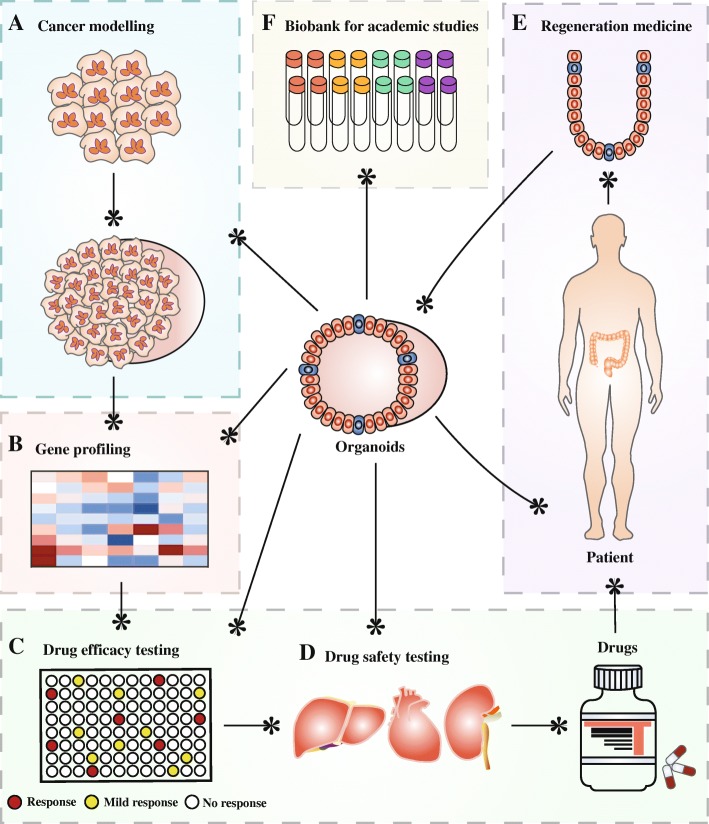
Potential applications of organoids in tumor modeling, drug development, and regeneration medicine. Organoid technology can be exploited to model human cancers (**a**), and gene-profiling analyses (**b**) of tumoroids and corresponding healthy organoids promote the identification of novel targeted therapies. Organoids can also promote the development of anti-tumor drugs, including efficacy testing (**c**) and toxicity testing (**d**). In addition, organoids can be a potential candidate in regeneration medicine for the replacement of irreversibly progressively diseased organs with healthy organoids (**e**). Besides, organoids can also be cryopreserved for academic studies (**f**)

These amazing 3D tissues in small scale are fabricated in the laboratory and resemble the parent organ in vivo in terms of structure and function. Three basic features are as follows: firstly, it contains multiple cell types of the in vivo counterpart; secondly, the cells organize similarly to the primary tissue; thirdly, it functions specifically to the parent organ [16]. This powerful technology bridges the conventional 2D in vitro models and in vivo models, and exerts great potential for clinical applications (Fig. 2), especially in cancer research [17]. Tumor modeling might be a pivotal branch of organoid technology [18, 19], including modeling infection-cancer development [20, 21], mutation-tumorigenesis processes [22, 23] and genetic carcinoma [24, 25]. Apart from cancer modeling, organoid technology also exerts enormous potential in evaluation of efficacy and toxicity of drugs [26], regeneration medicine [27, 28], and precision treatment [29, 30]. Until quite recently, organoids have been established successfully for multiple cancer types, including stomach cancer [26], colorectal cancer [31–33], liver cancer [34], pancreatic cancer [35, 36], prostate cancer [37], and breast cancer [38].

In this review, we outline a brief history of organoids, describe organoids of diverse cancer types, focus on the potential applications of this promising technology in oncology, and finally discuss the current limitations.

## The history of organoids

The notion that mammalian cells are inherently endowed with self-organizing capacity has long been widely known among researchers, and this ability has been employed to develop 3D cultures from primary tissues. Numerous types of culture systems have been reported in early studies [39–42], but no methods could achieve long-term culture and maintain the basic crypt-villus physiology. Encouragingly, the year 2009 witnessed the advancement of intestinal organoid culture system, a chief technological breakthrough in the SC field [43]. The novel culture system contained laminin-rich Matrigel replacing extracellular matrix (ECM) and growth factors including epidermal growth factor (EGF), Noggin, Wnt, and R-spondin. 3D mouse crypt structures in which continuously renewing epithelial layer exhausted apoptotic cells into a central lumen lined by crypt-like and villus-like sections were established in this 3D culture system, and these features remained when cultured for 8 months [43]. Subsequently, this culture system was adapted for the establishment of human intestinal organoids and other organ 3D architectures, such as the liver, stomach, and colon [44–46]. The organoid technology has become widely accepted in recent years, since these 3D cultures faithfully recapitulate the genotype, phenotype, and cellular behaviors of parent tissues [47].

Breast organoid cultures also experience a gradual evolution from the earliest attempts of in vitro cultivation of organ explants to the current relatively refined versions [48–50]. Mammary gland explants of from virgin mice could be cultivated in a serum-free medium, which consists of four major components: aldosterone, prolactin, insulin, and cortisol [48]. Through testing the mammary-derived growth inhibitor (MDGI) in mammary explants in vitro from mice at different development stages, it was demonstrated that MDGI expression was correlated with functional differentiation of normal mammary gland [48]. Next, mouse mammary gland cultivated in organ culture containing MRG protein showed a differentiated morphology with the upregulation of beta-casein [49]. Recently, it has been indicated that 3D cultures of breast cancer could more accurately model the structural and functional changes during the conversion from breast ductal carcinoma in situ to invasive carcinoma [50]. Up to now, breast cancer organoids have been efficiently established for studying breast cancer biology, and efforts are still in need for further improving culture conditions in order to overcome the current limitations.

Early in the 1980s, organotypic cultures have been employed to cultivate embryonic kidney, which allowed accurate manipulation of diversity developmental events in vivo in comparison with monolayer cell cultures [51]. However, the in vitro conditions led to metabolic changes, and it was difficult to realize long-term cultures because of the nutrition insufficiency-induced tissue damage [51]. When fetal murine metanephric tissues were isolated and incubated in serum-free medium, organotypic proximal tubular and glomerular epithelial differentiation were observed but without perfusion, urine production, and vascularization [52]. Quite recently, it was reported that host-derived vascularization formed in iPSC-derived kidney organoids in fully defined conditions without any exogenous vascular endothelial growth factor [53]. Progressive morphogenesis, including functional glomerular perfusion in function as well as connection to pre-existing vascular system, glomerular basement membrane, and fenestrated endothelial cells in structure, was observed in these organoids after transplanted under the kidney capsule [53].

Isolated brain cells, cultured in serum-free medium with classical hormones, EGF, fibroblast growth factor (FGF), attachment factors/basal membrane components, transport proteins, transferrin, albumin, vitamins, experienced morphological, bioelectrical, and biochemical differentiation [54–56]. During the past a few years, a variety of neural organoids have been established from ESCs or iPSCs in refined 3D culture systems which faithfully manipulated brain structures and some specific functions, including the whole brain [57] and sub-brain regions, such as hypothalamus [58], adenohypophysis [59], midbrain [60], cerebellum [61], and hippocampus [62].

## Establishment of cancer organoids

**Fig. 3:**
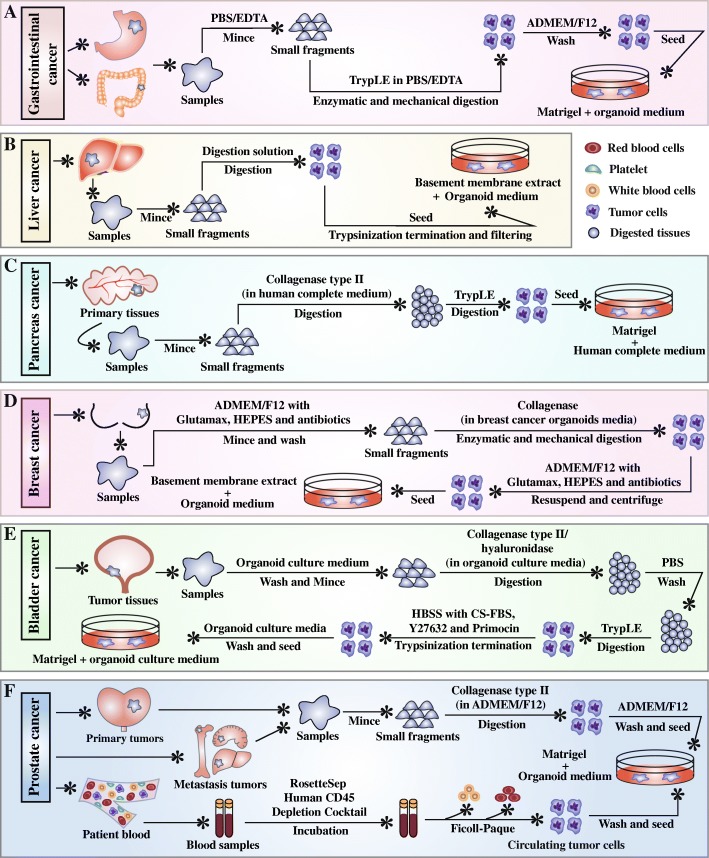
Flow charts for tumoroid establishment processes. Major steps during tumoroid establishment of gastrointestinal cancer (**a**), liver cancer (**b**), pancreatic cancer (**c**), breast cancer (**d**), bladder cancer (**e**), and prostate cancer (**f**) are shown

The poverty of in vitro tumor models that mimic the heterogenicity of human cancers has impeded the full understanding on tumor pathogenesis, therapeutic responses, and adverse reactions. The 3D organoid system draws researchers’ attention and has tremendous potential for modeling human cancers [63–65]. Major establishment procedures for each cancer organoid type were showed in Fig. 3. 3D culture system for organoid establishment consists of Matrigel or basement membrane extract as ECM substitutes and specific culture medium. Components in organoid culture medium majorly include advanced Dulbecco’s modified Eagle’s medium (ADMEM)/F12, penicillin/streptomycin, primocin, GlutaMAX, HEPES, B27, N2, EGF, FGF10, FGF7, hepatocyte growth factor (HGF), Wnt3A, Noggin, R-spondin-1, gastrin, prostaglandin E_2_, nicotinamide, neuregulin 1, N-acetylcysteine, Y27632 (a Rho kinase inhibitor), A-83-01 (a transforming growth factor-beta inhibitor), and SB202190 (a p38 inhibitor) (Table 1). There are minor differences in medium components among different tumoroid types [26, 34, 38, 66–68], shown in Table 2. Compared with conventional 2D cultures of cell lines, the most outstanding feature is the addition of ECM substitutes in 3D cultures.

**Table 1:** Growth factors and small molecule inhibitors applied in organoid cultures

	Function
Growth factors	
EGF	◆ A well-known growth factor for epithelial tissues; ◆ EGF, binding to EGF receptors, induces hyperplasic changes; ◆ EGF promotes tumor growth through stimulating the proliferation of cancer cells.
FGF10	◆ FGF10/FGF receptor 2IIIb axis is important for the organ development, including the stomach, liver, breast, and prostate; ◆ FGF10 promotes migration and invasion of pancreatic cancer cells and drives tumorigenesis of breast cancer;
FGF7	◆ FGF7/FGF receptor 2 signaling promotes growth, invasion, and migration of tumors.
HGF	◆ HGF/Met signaling promoted oncogenesis, tumor angiogenesis, tumor invasion of multiple tumor types;
Wnt	◆ A master regulator in regulation of cell development, proliferation, differentiation, adhesion, and polarity; ◆ The aberrant activation of Wnt signaling promotes carcinogenesis and progression of cancers.
Noggin	◆ An inhibitor of bone morphogenetic proteins that modulates cellular differentiation, proliferation, and apoptosis; ◆ Noggin promotes bone metastasis of some cancers and is associated with tumorigenesis of primary bone malignancies.
R-spondin-1	◆ The ligand of Lgr5 and a niche factor that is required for the self-renewal of stem cells and activates Wnt signaling; ◆ R-spondin-1facilitates the growth and metastasis of cancer cells.
Gastrin	◆ Gastrin stimulates tumor growth through promoting the proliferation and suppressing the apoptosis of cancer cells;
Prostaglandin E_2_	◆ Prostaglandin E_2_ promotes angiogenesis in gastric cancer through the up-regulation of vascular endothelial growth factor.
Nicotinamide	◆ Vitamin PP is a nutrient that is required for long-term culture of organoids.
Neuregulin 1	◆ It is a ligand of human EGF receptor tyrosine kinases-3 and -4; ◆ It is involved in mammary development and tumorigenesis.
Molecule inhibitors	
Y27632	◆ A Rho kinase inhibitor that effectively reduces the anoikis of dissociated stem cells; ◆ Y27632 improves culture media and promotes proliferation of tumor epithelial cells for long-term in vitro;
A-83-01	◆ A transforming growth factor-beta inhibitor; ◆ Transforming growth factor-beta inhibitor suppresses the proliferation of organoids;
SB202190	◆ It is a p38 inhibitor and suppresses the proliferation and migration of cancer cells; ◆ High concentration of SB202190 contributes to relatively lower efficiency of the establishment of breast tumoroids.

**Table 2:** Culture systems of multiple tumoroids

Tumoroid type	Culture components			Ref
	Extracellular matrix	Growth factors	Molecule inhibitors	
Stomach cancer	Matrigel (growth factor reduced)	ADMEM/F12, penicillin/streptomycin, L-glutamine, B27, N2, bovine serum albumin, EGF, Noggin, R-spondin-1, gastrin, FGF10, FGF-basic, Wnt3A, prostaglandin E_2_, and nicotinamide	A-83-01 Y27632 SB202190	[26]
Intestinal cancer				
Liver cancer	Basement membrane extract	Classical human liver organoid isolation medium: ADMEM/F12, penicillin/streptomycin, GlutaMAX, HEPES, B27 (without vitamin A), N2, N-acetylcysteine, nicotinamide, gastrin 1, EGF, FGF10, HGF, forskolin, R-spondin-1, Wnt3A, and Noggin Tumoroid-specific isolation medium: Classical human liver organoid isolation medium with the elimination of R-spondin-1, Wnt3A, and Noggin as well as addition of dexamethasone Human healthy liver-derived organoids expansion medium Classical human liver organoid isolation medium with the elimination of Y27632, Wnt3A, and Noggin	A-83-01 Y27632	[34]
Pancreatic cancer	Matrigel	ADMEM/F12, penicillin/streptomycin, GlutaMAX, HEPES, B27, N-acetylcysteine, EGF, R-spondin-1, gastrin 1, Wnt3A, Noggin, and FGF	A-83-01	[66]
Breast cancer	Basement membrane extract (reduced growth factor)	ADMEM/F12, penicillin/streptomycin, GlutaMAX, HEPES, B27, N-acetylcysteine, R-spondin-1, FGF7, FGF10, nicotinamide, Noggin, primocin, and neuregulin 1	A-83-01 Y27632	[38]
Bladder cancer	Matrigel	Hepatocyte media with EGF, FBS, GlutaMAX, and primocin	Y27632	[67]
Prostate cancer	Matrigel (growth factor reduced)	ADMEM, penicillin/streptomycin, primocin, GlutaMAX, B27, EGF, N-acetylcysteine, FGF10, FGF-basic, nicotinamide, testosterone, prostaglandin E_2_, Noggin, and R-spondin	A-83-01 SB202190	[68]

### Stomach cancer

Organoid technology has been applied to model gastric cancer [26, 69]. There are some subtle differences among studies in detailed manipulation. The proliferation rates of gastric cancer organoids were significantly higher than normal controls in vitro, and tumor growth of organoid engrafts in vivo was consistent with the expansion rates of corresponding organoids in vitro [69]. The organoids faithfully recapitulated important characteristics of the corresponding parent tumors as exemplified by architectures, the expression of typical gastric cancer markers including carcinoembryonal antigen, cadherin 17, cytokeratin 7 (KRT7), and periodic acid Schiff reaction [69]. These organoids harbored diverse mutations, which were prevalent in gastric cancer and could be detected in corresponding primary tumors, such as mutations in *mutL homolog 1*, *mutS homologs 6*, *phosphatidylinositol 3-kinase catalytic subunit*, *ERBB2*, and *TP53* [69].

### Intestinal cancer

Intestinal cancer organoids have been successfully developed in several studies [26, 31–33]. Sato T and colleagues demonstrated that colorectal cancer organoids responded diversely to Wnt3A/R-spondin-1, oxygen concentration, and SB202190 in organoid proliferation in consideration of the phenomenon that some tumoroids needed Wnt activators, some required hypoxia, and some showed growth suppression in reaction to SB212090 exposure [31]. Colorectal cancer organoids have been successfully propagated from different anatomical sites (right-sided, left-sided, and rectal tumors) and rare histological subtypes (mucinous adenocarcinoma and neuroendocrine carcinoma) [31]. Colorectal cancer organoids showed remarkable resemblance with the primary tumors in the aspects of histological subtypes, differentiation hierarchies, mutational landscape, and transcriptomic profiling [26, 31]. It was noted that colorectal cancer organoids in combination with an orthotopic transplantation system could more accurately model tumor formation and liver metastasis in the native colon environment [70]. Proteomic analyses on colorectal cancer organoids showed each organoid from distinct patients harbored different proteomic profiles, which signifies that specific organoid proteome profile from patients can guide precision management [71].

### Liver cancer

Human liver cancer organoids have been established in several studies [72]. Primary liver cancer organoids of three major types including hepatocellular carcinoma (HCC), cholangiocarcinoma (CC), and combined hepatocellular-CC (CHC) have been successfully developed in specific isolated medium and passaged in expansion medium [34]. Specific isolated medium used during the establishment of liver cancer organoids includes two types: classical human liver organoid isolation medium and tumoroid-specific isolation medium [34]. Some organoids needed tumoroid-specific isolation medium, while some other organoids required classical isolation medium [34]. It was observed that one CC organoid only grew in classical human liver organoid isolation medium due to the need of R-spondin-1 for growth [34]. Y27632 is only required during the first 2–3 weeks of culture. At histological level, these primary liver cancer organoids recapitulated their parent tumors to a great degree even after long-term expansion [34]. The organoids of HCC and CHC were solid architectures filled with HCCs, in which a histological characteristic of HCC (pseudoglandular rosettes) was observed [34]. Just as found in patients’ tissues, it was also noted that CC tumoroids contained a great many glandular regions with cancer cells, which invaded the lumen and grew in a cribriform manner [34]. For expression profile, alfa-fetoprotein and glypican-3, markers of HCC, were upregulated in HCC tumoroids but the levels of CC markers remained low [34]. Conversely, CC markers (epithelial cell adhesion molecules, KRT19, and S100 Calcium Binding Protein A11 were enhanced in CC organoids but HCC markers were remarkably downregulated [34]. For transcriptional level, these organoids faithfully recapitulated transcriptomic alterations, which were identified in corresponding original tissues [34].

### Pancreatic cancer

Pancreatic tumor organoids have been successfully established in a flurry of studies [36, 66, 73]. For long-term maintenance and enrichment of *KRAS*-mutant pancreatic ductal adenocarcinoma (PDAC) organoids, serum and EGF were eliminated from the culture medium [36]. For the organoids that were sensitive to the removal of EGF, an inhibitor of murine double minute 2 Nutlin3 or Noggin elimination could be employed to select possible existing organoids with *TP53* or *SMAD4*-mutants, respectively [36]. Driver-gene alterations including *KRAS*, *cyclin-dependent kinase inhibitor 2A*, *TP53*, and *SMAD4*, which are common in human pancreatic carcinoma, were detected in corresponding organoids. When transplanted into mice, the organoids formed tumors in vivo like the derived PDAC [36]. Optical metabolic imaging of PDAC organoids is quite sensitive to metabolic changes induced by anti-cancer drugs. The combination of this nondestructive method and cancer organoid platform help better monitoring of dynamic drug response for patients in vitro [74].

### Breast cancer

Breast cancer organoid models have been successfully achieved to study breast carcinoma biology [38, 75, 76]. Hans Clevers, et al. highlighted that (1) neuregulin 1 was an essential element for efficient generation and long-term expansion for breast cancer organoids; (2) Wnt3A was not essential for culture conditions; (3) EGF was a double-edged sword for low concentration impeding proliferation and high concentration leading to organoid sinking and gradual loss of 3D organization; (4) SB202190 at high concentration was detrimental to effective establishment of breast cancer organoids [38]. The breast cancer organoid lines were consistent with the parent tumors in morphology, histopathology, hormone receptor status, human epidermal growth factor receptor 2 (Her2) status, mutational landscape, and DNA CNAs [38]. Organoids represent a valuable tool for evaluating local tumor invasion of breast cancer, which is the basis for distant metastasis and involves the interactions between tumor, ECM, and stromal cells [75].

### Bladder cancer

The culture system of bladder cancer organoids has been reported in many studies [67, 77]. A biobank of patient-derived bladder cancer organoids has been established by Suk Hyung Lee and colleagues, who reported a well-defined culture protocol for propagation of bladder cancer organoids [67]. Histological analysis demonstrated the remarkable similarity between these organoids and the corresponding derived tumors [67]. In terms of the mutational profiles for 468 tumor-related genes, high concordance was observed between bladder cancer organoids and their parental tumors [67]. However, there were some genomic changes in organoids, which accompanied with cancer evolution in culture [67]. According to the deep sequencing analysis, some mutations were lost or gained during the continuous process in organoid cultures [67]. Using bladder tumoroids as a platform, drug response was partly associated with mutational profiles, signifying the feasibility that bladder tumor organoids derived from patients can be employed to predict treatment response and guide personalized therapies for each individual patient [67].

### Prostate cancer

Prostate cancer organoids from patients have been reported in multiple studies [37, 68, 78]. Dong Gao’s group provided a detailed protocol for the metastatic prostate cancer organoid establishment from metastatic tumor cells and circulating tumor cells [37]. A diversity of characteristic copy number alterations (CNAs) in prostate cancer were detected in the prostate tumoroid lines, including deletions of SHQ1, transmembrane protease, serine 2/erythroblast transformation-specific-related gene and phosphatase and tensin homolog (PTEN) as well as the amplification of androgen receptor (AR) [37]. Furthermore, mutation profile detected in organoid lines overlaid the prevalent mutations in prostate cancer, such as mutations in TP53, forkhead box A1, phosphoinositide-3-kinase regulatory subunit 1 (PIK3R1), alpha thalassemia/mental retardation syndrome X-linked, checkpoint kinase 2, KDM4C, KDM4D, and MLL2 [37]. When transplanted into severe combined immunodeficient mice, organoid lines displayed histological patterns found in parent tumors [37]. The 3D co-cultures of bone stroma cells and prostate cancer cells not only induced cytogenetic and gene expression changes in stromal cells but also fueled growth and metastasis of prostate tumoroids, which indicated the co-evolution of cancer and stroma as well as the significance of tumor-stroma interaction [79].

### Other cancer types

Organoids of other cancer types have also been faithfully established, such as CC [26], thyroid cancer [80], ovarian cancer (OC) [81], and brain cancer [82]. CC organoids derived from human metastatic CC biopsies retained rearrangements of fibroblast growth factor receptor 2 that parent tumors harbored [26]. Mouse models of poorly differentiated thyroid tumors has been established through the transplantation of the thyroid organoids with enhanced expression of oncogene neuroblastoma *RAS* derived from mouse with *P53* knockout [80]. In addition, OC cell lines from patients were planted on Matrigel in cancer SC medium containing Gentamicin, Fungizone, and Y27632, and formed organoids with the expression of tumor marker carbohydrate antigen 125 [81]. The infiltration capacity of glioblastoma multiforme cell into healthy brain parenchyma partly accounts for that high-grade of this tumor type cannot benefit much from surgical management [82]. Human glioblastoma multiforme spheroids could spontaneously infiltrate early-stage brain organoids and form hybrid organoids, demonstrating an invasive tumor phenotype and helping explore anti-invasion strategies for this refractory disease [82].

However, organoid models of some cancer types have not been reported as exemplified by lung cancer. Lung normal organoids can be developed from basal cells derived from trachea or large airways or even nasal epithelium, commonly containing TRP63^+^and KRT5^+^ basal cells, secretory goblet cells, and functional multiciliated cells [83, 84]. Through clustered regularly interspersed short palindromic repeats (CRISPR)/CRISPR-associated protein 9 (Cas9) gene editing technology, organoid can be employed as a platform to identify genes that modulate vital airway functions, such as selective permeability, barrier formation, fluid transport, innate immunity, and ciliogenesis [85, 86]. According to these findings, we can suppose that oncogene-activated mutations introduced by CRISPR/Cas9 might drive tumorigenesis in primary normal lung organoids. Further efforts are in need for application of organoid technology in lung cancer.

## Organoid in cancer modeling

Some infectious pathogens are identified to be significant risk factors of cancer, such as *Helicobacter pylori* in gastric cancer, *Salmonella enterica* in gallbladder carcinoma, hepatitis virus in HCC, and Epstein-Barr virus (EBV) in gastric cancer, nasopharyngeal carcinoma, and lymphoma. However, there is still a lack of extensive understanding of the direct relationships and causal mechanisms between the infectious pathogens and corresponding cancers. Organoids can serve as a potential excellent model for studying these processes through co-culture systems with different pathogens. Neefjes J and colleagues employed co-cultures of murine-derived genetically predisposed gallbladder organoids and *Salmonella enterica* to explore the epidemiological association between gallbladder carcinoma and *Salmonella Typhi* infection, and supported that *Salmonella enterica* triggered and maintained malignant transformation accompanied by *TP53* mutations and *c-Myc* amplification through *Salmonella enterica* effectors-induced activation of mitogen-activated protein kinase and AKT pathways [20]. Besides, viral infectious organoid models can also be established as exemplified by intestinal organoids with rotavirus infection [21], indicating that the virus-tumor relationship can also be simulated by co-culture systems, such as hepatitis virus versus liver cancer and EBV versus nasopharyngeal carcinoma. Modeling of the transition from infection to tumor formation and progression of organoids might help to reveal pathogenic mechanisms and find potential anti-tumor targets during this process.

Cancers occur on the genetic basis of sequential accumulation of mutations, signifying that it is pivotal to throw light upon the mutational processes during homeostasis and tumorigenesis. Knowledge of original mutation profile has been demonstrated to be of importance [22], for which healthy organoids provide a platform. Whole genome sequencing on human colon organoids with knockout of DNA repair genes through CRISPA-Cas9 technology revealed that the deficiency in mismatch repair genes contributed to mutation accumulation through replication errors, and deficiency in the cancer-predisposition gene DNA glycosylase led to mutation profile previously noted in cancer patients [23]. In addition, understanding of heterogeneous mutational signatures underlying tumor progression is also of great significance, which can also be prompted by organoid technology. Remarkably increased mutation rates and acquisition of new mutational profile were observed during development of colorectal tumoroids, and the diverse contributions of mutational processes in different regions of the same tumor were demonstrated by Roerink SF and colleagues [87]. It is interesting and feasible to employ organoid platform to evaluate the impact of drugs and irradiation on mutation profiles of cancer and normal cells as well as explore the mutational differences between sensitive and resistant organoids towards treatments.

Genetic cancer modeling is another paramount potential application of tumoroids [24, 25, 88, 89]. The conversion from healthy human intestinal organoids to colorectal progressive tumoroids has been achieved through the introduction of a set of common driver mutations in colorectal cancer via CRISPR-Cas9 gene editing technology, indicating tumor growth as a consequence of cancer driver mutations was independent of SC niche factors and identifying loss of *adenomatosis polyposis coli* (*APC*) and *TP53* as pivotal contributors for chromosome instability and aneuploidy [24, 90]. Using organoid models, it was demonstrated that *ring finger protein 43* mutations positively regulated Wnt-β-catenin signaling in human serrated colon adenoma [91], and loss of mutations in *caudal type homeobox2* and *BRAF*^V600E^ synergistically drove progression of serrated colorectal cancer [89]. Organoids facilitate better understanding of tumor initiation and progression of cancers at the genetic level.

## Organoids in drug development

During the past decades, numerous anti-cancer drugs developed from screening on conventional 2D culture of large standard cell lines failed in clinical studies [92, 93]. For most cytotoxic agents, broad activity was observed across tumor cell lines, but clinical efficiency noted in patients was in more limited settings [93]. Voskoglou-Nomikos T evaluated whether in vitro cell lines were reliable in predicting clinical utility. The results showed that in vitro cell line model was predictive for non-small cell lung cancer under the disease-oriented approach, but not for colon cancer [94]. Since cancer organoids are near-physiological architectures, retain specific functions of the parent tumors and can faithfully recapitulate drug responses, the organoid technology fills the gap between drug screening based on classical 2D cell lines and clinical trials. Numerous studies have demonstrated that organoid can serve as an excellent model for evaluating specific responses of cancer patients [26, 69, 81, 95, 96]. Besides, it also can be an extraordinary alternative to explore the detailed causal epigenetic and genetic alterations underlying drug resistance [97]. Several organoid biobanks of cancers so far have been established for the purposes of identifying and testing novel drugs [37, 38, 98], and healthy organoids can be utilized to test toxicology.

### Drug efficacy testing

Recently, metastatic gastrointestinal cancer (colorectal cancer and gastroesophageal cancer) organoids derived from patients have been established and employed to identify whether organoids can forecast treatment response among patients. In this study, a wide spectrum of anti-tumor drugs, including used in clinical practice and currently in phases of clinical trials, were enrolled for testing drug sensitivity [26]. The results reflected that organoids cancer faithfully recapitulated treatment responses of gastrointestinal cancers with high sensitivity (100%), specificity (93%), positive predictive value (88%), and negative predictive value (100%) in predicting response to chemotherapy in patients [26]. For instance, there was a remarkable association between retinoblastoma 1 amplification and the sensitivity of tumor organoids to cyclin dependent kinase 4/6 inhibitor palbociclib, which was in line with previously published data [26, 99]. Another example was that patient-derived organoids with *BRAF*^V600E^ mutation exhibited dramatically reduced viability but no differences in apoptosis after the exposure of the *BRAF* inhibitor vemurafenib in comparison with the organoids with no mutations in *BRAF* gene, which was consistent with the ineffectiveness of monotherapy with *BRAF* inhibitors in metastatic colorectal cancer [26]. By conducting drug screening on human gastric cancer organoids, Therese Seidlitz and colleagues identified organoids recapitulated the divergent responses to conventional chemotherapeutics, including 5-fluorouracil (5-FU), irinotecan, epirubicin, oxaliplantin, and docetaxel [69]. Furthermore, these organoid lines can be employed to test not only the efficacy of a known mutation-targeted therapy for an individual patient but also the effectiveness of treatment on unknown mutations, as exemplified by trastuzumab treatment for *ERBB2* amplifications/*ERBB2* mutations and imatinib treatment for an unknown mutation in exon 3 of the *KIT* receptor [69].

A panel of human colorectal cancer organoids has been assembled for assessing mutation-targeted inhibitors and drug combination therapy, including irreversible epidermal growth factor receptor/Her2 inhibitor afatinib, MEK inhibitor selumetinib, and ERK inhibitor SCH772984 [100]. The results reflected that both the combinations of afatinib plus selumetinib and SCH772984 plus selumetinib significantly inhibited growth of *RAS*-mutant tumor organoids with obvious cell cycle block but no impact on cell death. After these drugs were withdrawn, tumor cells could restore proliferation activity, which might hamper the effectiveness of the combination therapy among patients with RAS-mutant colorectal cancer [100]. However, the combination of a preclinical B-cell lymphoma 2 (BCL-2)/BCL-xL inhibitor navitoclax, afatinib, and selumetinib potently promoted cell death in comparison with monotherapy of these drugs, indicating a possible alternative treatment strategy [100].

Huch M, et al. has propagated primary liver tumoroids, which faithfully recapitulated histology, expression patterns and genetic alterations of corresponding original tumors [34]. A total of 29 anti-cancer drugs were enrolled in the proof-of-concept testing of drug sensitivity using organoid model, and the results indicated that these tumoroids facilitated identification of drug sensitivity in individual patient. Intriguingly, it was identified that ERK signaling could be a potential therapeutic target for primary liver cancer patients [34].

A living biobank of primary breast cancer organoids and metastatic breast cancer organoids can also be employed as an excellent platform for drug screening, supported by that responses to afatinib or tamoxifen of organoids showed remarkably similarity to patients [38]. As another example, standard OC cells from patients were cultured to differentiate into organoids [81]. The responses to multiple OC drugs and the association with genomic alterations in organoids were assessed through *DeathPro* assay for improving drug screening [81]. A diversity of drug responses were observed in OC organoids and drug effects in organoids resembled the findings in clinical trials [81]. For instance, a majority of OC patients failed to response to paclitaxel, and the addition of paclitaxel to carboplatin did not refine efficacy in comparison to carboplatin monotherapy [81]. Compared with 2D cultures, the responses to drugs of organoids were more similar to the parent tumors. Dasatinib, to which recurrent OC is resistant at clinical phase II, was also ineffective in 3D culture but effective in 2D culture [81].

Because of the extraordinary recapitulation of responses to drugs for original tumors in vivo, prostate cancer organoid lines have also been exploited to help the screening of anti-cancer drugs [37]. For instance, *AR*-amplified prostate cancer organoids were exquisitely sensitive to the AR inhibitor enzalutamide, while *AR*-negative prostate cancer organoids responded to this drug in an opposite manner [37]. Besides, prostate cancer organoid lines harboring both *PTEN* loss and *PIK3R1* mutation were sensitive to everolinus and BKM120 [37].

### Pharmacokinetic

Organoids technology can also be employed in pharmacokinetic testing, which is a pivotal thing during drug development. Human iPSCs-derived intestinal organoids have been generated through appropriate methods with a variety of intestinal cells [101], and these organoids were endowed with pharmacokinetic function [101]. In the condition of some small-molecular compounds, organoids expressed drug transporters, efflux transport activity, and the activation of drug-metabolizing enzyme cytochrome P450 [101]. The results indicated that these organoids could be employed for pharmacokinetic assessment in drug development [101].

### Drug toxicity testing

Another major advantage of organoid technology in drug development is that normal organoids can be generated and exploited for screening of drugs which exclusively target tumor cells without harming healthy cells. Intolerant side effects majorly lead to drug failure in clinical trials, including hepatotoxicity, cardiotoxicity, and nephrotoxicity. Hepatic organoid represents an extraordinary model for hepatotoxicity testing of experimental compounds [102–104]. Drug-related hepatotoxicity is mostly mediated through cytochrome P450 enzymes, which is inspiringly observed in hepatic organoids at near-physiological levels [104, 105]. Cardiac adverse effects such as arrhythmias and cardiotoxic effects can also be tested in 3D cultures [96, 106]. Besides, kidney organoids has also been employed for toxicological research [107].

## Immunotherapy

Immunotherapy, which is among the chief novel and promising strategies, employs the patient’s own immune system to kill tumor cells. A prerequisite for immunotherapy is that malignant cells exhibit sufficient immunogenicity to trigger adequate immune response [108, 109]. Mutational status of cancer cells, which contribute to neo-antigens production, is responsible for immune responses [109, 110]. However, the intensity of immune response induced by neo-antigens of carcinoma is insufficient, which can be addressed through activating and expanding immune cells in vitro for in vivo application in patients.

Multiple studies have brought new hope for the application of organoid technology in immunotherapy, as exemplified by functional maintenance of intraepithelial lymphocytes being co-cultured with mouse intestinal organoids at the presence of interleukin-2 (IL-2), IL7, and IL-15 in the culture medium [111]. Another example is that the short-term maintenance of CD45-positive lymphocytes can be achieved through co-culture with patient-derived organoids of air-liquid interface tumors [112]. Encouragingly, co-cultures of Vδ2^+^ T lymphocytes and organoids of primary human breast epithelial have been developed successfully, and these T lymphocytes could potently eradicate triple-negative breast cancer cells [113]. These findings signify the possibility that T lymphocytes from healthy blood donors can be expanded and activated with organoids and subsequently utilized to treat patients, and the possibility that the cytotoxic effects of healthy donor-derived T cells on patient-derived tumoroids can be tested in vitro.

## Personalized medicine

Personalized medicine, also called precision medicine, aims to identify effective treatment strategies for each patient through better characterization of diseases at molecular and pharmacogenomics levels. As an excellent minute incarnation of an in vivo organ, organoids are superior to conventional models, because this easily established model can better recapitulate in vivo characteristics in phenotype, genotype, and specific functions as well as physiological and pathological changes even after many generations. Organoids are endowed with enormous potential to identify the feasible optimized treatment strategy for the individual patient [29, 30, 114, 115].

Rubin MA and colleagues applied the organoid platform to identify the optimized combination therapy options for some cancer types as exemplified by uterine carcinosarcoma and endometrial adenocarcinoma harboring similar driver mutations in *PIK3 catalytic subunit alpha* and *PTEN* [29]. The uterine carcinosarcoma organoid receiving combination treatment of vorinostat and buparlisib showed strongest inhibition in comparison with other combination strategies, while the combination of buparlisib andolaparib was among the most effective strategies for the endometrial adenocarcinoma organoid [29].

Another example was that the *KRAS* and *TP53*-mutant organoid of stage IV colorectal cancer only showed notable response to trametinib, and the combination of trametinib and celecoxib was among the chief strongly effective combinational options [29]. Besides, it was also demonstrated that the novel combination of afatinib and histone deacetylase inhibitors contributed to dramatically enhanced growth suppression of colorectal tumoroids with *APC* mutations, even greater than the standard FOLFOX (oxaliplatin, FU and leucovorin) regimen did [29]. In addition, drug screening was also conducted on human colorectal organoids from patients, containing many cancer SCs and being resistant to 5-FU and irinotecan [116]. Organoids treated with hedgehog signal inhibitors (AY9944 and GANT61) exhibited reduced cell viability with downregulation of c-Myc, CD44, and Nanog [116], and organoids treated with the combination of AY9944 or GANT61 with 5-FU or irinotecan showed impaired cell viability in comparison to each drug alone [116]. These results reflected that inhibitors of hedgehog signaling could serve as an effective combinational candidate for the treatment of 5-FU or irinotecan-resistant colorectal tumors [116]. Based on the phenomenon that anaplastic lymphoma kinase (ALK) mutation (F1174C) promoted growth and upregulated the expression of neuroendocrine marker neuron-specific enolase in the organoids of prostate small cell carcinoma, alectinic showed more significant effects than crizotinibin terms of inhibiting ALKF1174C-expressing cell expansion [117].

Photodynamic therapy, known as a light-activated cancer therapy, supplements conventional chemotherapies and brings clinical promise for pancreatic cancer treatment [118]. As observed in organoids of metastatic pancreatic carcinoma, intelligent combination of oxaliplatin and neoadjuvant photodynamic therapy exhibited remarkably enhanced anti-tumor efficacy in comparison with any therapy alone, without augment of toxicity [118].

Although it is still in an immature stage of organoid technology in personalized medicine, further efforts can refine this model and broaden horizon in personalized medicine in replacement for conventional “one-size-fits-all” treatments.

## Current limitations

Although organoids have a wide range of potential applications, the current version still represents a somewhat rough model, and researchers still grapple with obstacles of this technology. Firstly, organoids are imperfect reproductions. The “tissues in a dish” comprise only epithelial layer without native microenvironment including surrounding mesenchyme, immune cells, nervous system, or muscular layer [81]. Possible solutions to this limitation are to further refine organotypic culture system or to co-culture with additional cellular elements such as immune cells, stromal cells, or neural cells, as exemplified by iPSC-derived intestinal organoids containing a functional nervous system [119] and co-culture of PDAC organoids with mouse pancreatic stellate cells which differentiated into cancer-related fibroblasts [120]. In spite of these encouraging findings, an immune microenvironment around a tumor is difficult to be modeled. Immune niche of tumors is a complicated system composed of diverse immune cells including cytotoxic lymphocytes, tumor infiltrating dendritic cells, regulatory T cells, tumor-associated macrophage, and myeloid-derived suppressor cells, and tumor immune microenvironment is in dynamic changes, and there may be differences between different tumor types as well as individual patients. Secondly, fully maturation is an obstacle required to be tackled, which might affect the therapeutic potential. Thirdly, some organoid lines still cannot be expanded for long term, which could be disposed through improvement of culture medium. Fourthly, cancer organoids tend to grow more slowly than corresponding organoids from normal epithelial, thus probably contributing to the outgrowth of tumor organoids by contaminating normal epithelial cells. This problem might be addressed through improving the tissue extraction process to minimize the contaminating normal cells. Fifthly, current organoids are majorly derived from epithelium, and further investigation of cultures of non-epithelial organoids is needed, taking the recent advances in establishment of organoids induced from primary glioblastoma as an example. Lastly, the growth factors or small molecular inhibitors in culture medium may have significant effects on gene expression and signaling pathways in organoids, and may affect drug sensitivity. Further efforts are in need for addressing this problem.

## Conclusion

In spite of these limitations, the exciting and promising organoid technology holds enormous potential to more accurately model human tumors. Up to now, highly efficient establishment of organoids has been achieved from both normal and malignant tissues. Using these amazing 3D cultures, both drug screening and personalized medicine can be prompted dramatically to better predict drug responses and guide optimized therapy strategies for an individual patient. Future efforts will doubtless bring this novel technique closer to clinical practice.

## Abbreviations

2D: Two-dimensional
3D: Three-dimensional
5-FU: 5-fluorouracil
ADMEM: Advanced Dulbecco’s modified Eagle’s medium
ALK: Anaplastic lymphoma kinase
APC: Adenomatosis polyposis coli
AR: Androgen receptor
BCL-2: B-cell lymphoma 2
Cas9: CRISPR-associated protein 9
CC: Cholangiocarcinoma
CD45: Cluster of differentiation 45
CHC: Combined hepatocellular-cholangiocarcinoma
CNAs: Copy number alterations
CRISPR: Clustered regularly interspersed short palindromic repeats
EBV: Epstein-Barr virus
ECM: Extracellular matrix
EGF: Epidermal growth factor
ESCs: Embryonic stem cells
FBS: Fetal bovine serum
FGF10: Fibroblast growth factor 10
HBSS: Hank’s balanced salt solution
HCC: Hepatocellular carcinoma
Her2: Human epidermal growth factor receptor 2
HGF: Hepatocyte growth factor
IL-2: Interleukin-2
iPSCs: Induced pluripotent stem cells
KRT7: Cytokeratin 7
MDGI: Mammary-derived growth inhibitor
OC: Ovarian cancer
PBS: Phosphate-buffered saline
PDAC: Pancreatic ductal adenocarcinoma
PDTXs: Patient-derived tumor xenografts
PIK3R1: Phosphoinositide-3-kinase regulatory subunit 1
PTEN: Phosphatase and tensin homolog
SCs: Stem cells
